# Effects of in vitro fertilization and embryo culture on TRP53 and Bax expression in B6 mouse embryos

**DOI:** 10.1186/1477-7827-4-61

**Published:** 2006-11-21

**Authors:** Vashe Chandrakanthan, Aiqing Li, Omar Chami, Christopher O'Neill

**Affiliations:** 1 Discipline of Physiology, University of Sydney, Royal North Shore Hospital, St Leonards, NSW 2065, Australia; 2 Discipline of Medicine, University of Sydney, Royal North Shore Hospital, St Leonards, NSW 2065, Australia

## Abstract

In the mouse, embryo culture results in a characteristic phenotype of retarded embryo preimplantation development and reduced numbers of cells within embryos. The expression of TRP53 is central to the regulation of the cell's capacity to proliferate and survive. In this study we found that Trp53 mRNA is expressed throughout the preimplantation stage of development. Levels of TRP53 protein expression were low during the cleavage stages and increased at the morula and blastocyst stages in B6 embryos collected from the reproductive tract. Embryos collected at the zygote stage and cultured for 96 h also showed low levels of TRP53 expression at precompaction stages. There were higher levels of TRP53 in cultured morula and the level in cultured blastocysts was clearly increased above blastocysts collected directly from the uterus. Immunolocalization of TRP53 showed that its increased expression in cultured blastocysts corresponded with a marked accumulation of TRP53 within the nuclei of embryonic cells. This pattern of expression was enhanced in embryos produced by in vitro fertilization and subjected to culture. The TRP53 was transcriptionally active since culture also induced increased expression of Bax, yet this did not occur in embryos lacking Trp53 (Trp53-/-). The rate of development of Trp53-/- zygotes to the blastocyst stage was not different to wildtype controls when embryos were cultured in groups of ten but was significantly faster when cultured individually. The results show that zygote culture resulted in the accumulation of transcription activity of TRP53 in the resulting blastocysts. This accounts for the adverse effects of culture of embryos individually, but does not appear to be the sole cause of the retarded preimplantation stage growth phenotype associated with culture in vitro.

## Introduction

Assisted reproductive technologies (ART; which includes techniques such as *in vitro *fertilization (IVF), intracytoplasmic sperm injection (ICSI), embryo cryopreservation and embryo donation) are now central to the treatment of infertility in humans. These techniques are successful treatments, yet an individual embryo produced by these methods has less than a 50% chance of forming a viable neonate. Much of the loss of embryo viability occurs in the pre- and peri-implantation phases.

The use of animal models, particularly the mouse, has been important for the development of embryo culture procedures. In the mouse it is well known that embryo manipulation and culture results in a characteristic phenotype of slow embryo development *in vitro*. After several days in culture embryos have typically fewer cells and more cells within embryos undergoing death, than corresponding stage embryos collected from the reproductive tract [1-3]. This phenotype of impaired development is particularly severe in a range of inbred lines, such as B6 [4]. Such lines provide an attractive model for identifying the possible causative mechanisms.

A number of cellular stressors induced by the culture environment have been identified, and include growth and survival factor deprivation [3,5], metabolic and substrate imbalances [6,7], and oxidative stress [8]. In somatic cells all these stresses are capable of activating the TRP53 stress response pathway. Increased TRP53 expression is a response to a wide range of genotoxic and non-genotoxic stresses [9-11]. TRP53 is a transcription factor that has many functions [10] including a reduction in the rate of cycle-cell progression (for example, by the induction of cyclin-dependent kinase inhibitors such as p21^Waf1/Cip1^) or induction of cell death (by the synthesis of pro-death mediators, such as Bax).

Mouse [12] and human embryos [13] express *Trp53 *mRNA. In human embryos produced by IVF its expression was higher in embryos with poor morphology following culture, as assessed by the degree of cytoplasmic fragmentation [14]. There is much anecdotal evidence that transgenic over-expression of *Trp53 *is incompatible with early mouse embryo development. Furthermore, increased TRP53 activity (due to the deletion of *Mdm2 *[15-17]) also results in early mouse embryonic lethality. Diabetes-induced early embryopathy was partially ameliorated by *Trp53 *deletion in a mouse model [18].

This study examined the hypothesis that IVF and culture of embryos caused increased TRP53 expression of transcriptionally active TRP53 in the mouse preimplantation embryo.

## Materials and methods

### Animals

The use of animals was in accordance with the Australian Code of Practice for the Care and Use of Animals for Scientific Purpose and was approved by the Institutional Animal Care and Ethics Committee. C57BL6J (B6), *Trp53*^-/- ^and *Trp53*^+/+ ^(B6.129S2-Trp53^tm1Tyj ^strain extensively backcrossed with B6) were used in experiments. All animals were housed and bred in the Gore Hill Research Laboratory, St Leonards, NSW, Australia. All animals were under 12 h light: 12 h dark cycle and had access to food and water *ad libitum*. Four to eight week old females were superovulated by intraperitoneal injection of 10 IU equine chorionic gonadotrophin (Folligon, Intervet International, Boxmeer, The Netherlands) followed 48 h later by 10 IU human chorionic gonadotrophin (hCG, Chorulon, Intervet). Females were paired with males of proven fertility. Pregnancy was confirmed by the presence of a copulation plug the following morning (day 1).

### Mouse embryo collection and culture

In most experiments cumulus masses or embryos were flushed from the reproductive tract with HEPES-buffered modified human tubal fluid medium and cultured in modified human tubal fluid medium (mod-HTF) [5]. All components of the media were tissue culture grade (Sigma Chemical Company, St Louis, MO) and contained 3 mg bovine serum albumin/mL unless otherwise stated (CSL Ltd., Melbourne, Vic., Australia). Fertilization *in vitro *(IVF) was performed as previously described [19]. Briefly, epididymides from males of proven fertility were punctured and the sperm squeezed into medium and allowed to disperse in mod-HTF media for 40 minutes. Motile sperm (0.5 × 10^6^) was added to groups of oocytes within their cumulus masses. Fertilization rate was assessed at 5–6 h and all fertilized oocytes transferred to mod-HTF media. Fresh zygotes were collected 20–21 h after hCG and freed from their cumulus cells by brief exposure to 300 IU hyaluronidase (Sigma) in HEPES-buffered mod-HTF. Embryos were recovered in minimal volume and assigned to various treatments as required in mod-HTF. Embryos were cultured in 10 μL volumes in 60-well HLA plates (LUX 5260, Nunc, Naperville, IL) overlaid by approximately 2 mm of heavy paraffin oil (Sigma). Embryos were cultured individually or in groups of 10. Culture was at 37°C in 5% CO_2 _for the periods indicated in individual experiments. The developmental stage and morphology of embryos was assessed by visualizing the embryos with an inverted phase contrasted microscope (Nikon Diaphot, Japan) at 24 h interval after zygote collection. Fresh blastocysts were collected by flushing each uterine horn with 1 mL of HEPES buffered mod-HTF.

### Cell lines

Two cell lines were used as positive control material. F9 (mouse choriocarcinoma) and T47D (estrogen receptor-positive breast cancer cell) cell lines were routinely grown in DMEM supplemented with 10% fetal calf serum, 0.1 IU/mL insulin, 2 mM glutamine, 100 IU/*mL *penicillin, and 100 μg/*mL *streptomycin in a humidified atmosphere containing 5% CO_2 _and 95% air at 37 C. The cells were grown to confluence and cells collected for analysis, washed three times in PBS, counted and then used a indicated.

### Reverse transcriptase polymerase transcription factor (RTPCR)

RTPCR was performed as previously described [20], briefly embryos were collected fresh from the reproductive tract. The embryos were washed 3 times in cold PBS (Ca^2+^- Mg^2+^-free Dubbecco's phosphate buffer saline (Sigma)) and then transferred in a minimal volume to 30 μl of PCR Gold buffer (50 mM KCl, 15 mM Tris-HCl, pH 8.0) in diethyl pyrocarbonate (DEPC, Sigma) treated MilliQ water containing 1 IU RNAse inhibitor (Applied Biosystems, Lincoln Centre Drive, Foster City, CA). The embryos were lyzed by 3 cycles of freezing in liquid nitrogen and thawing (with vortexing) and subject to RTPCR. Reverse transcription was in 12.5 U of murine leukemia virus reverse transcriptase (MuLV), 1 U RNase inhibitor, 4 mM MgCl2, 50 mM KCl, 15 mM Tris-HCl pH 8.0, 0.5 mM dNTPs (Applied Biosystems), 1.5 μM allele specific reverse primer. The reactions were incubated for 10 min in room temperature, 30 min at 42°C and 2 min at 99°C. Two negative controls were included: no reverse transcription enzyme; and no template to test for extraneous DNA or RNA contaminations, respectively. PCR reaction specific products included 3 μl cDNA template (equivalent to 0.3 embryo), 1.5 μL Amplitaq Gold DNA polymerase, 4 mM MgCl2, 50 mM KCl, 15 mM Tris-HCl pH 8.0, 0.5 mM dNTP (Applied Biosystems) and 5% (v/v) DMSO (Sigma), 1.5 μM each of gene specific primers. The reactions were incubated for 10 min at 94°C and 40 cycles of 15 seconds at 94°C and 1 min at 58°C in a Corbett Thermal Reactor. PCR reaction products were analyzed by electrophoresis on 2% (w/v) agarose gel stained with ethidium bromide to visualize PCR products on UV transluminator. Fragments were verified by size and representative samples were had sequence analyzed (SUPAMAC, Redfern, NSW, Australia).

Primers were obtained from Sigma-Genosys (Sigma). β-Actin was used as a positive control The sequence of oligonucleotide primers and the product size were as follows: β*-Actin *(Accession number MMACTBR) 5'-CGTGGGCCGCCCTAGGCACCA, 3'-TTGGCCTTAGGGTTCAGGGGG, 243 bp. *Trp53 *5' – GGAGTCTTCCAGTGTGATGAT 3'-GGGACAGCCAAGTCTGTTATG 429 bp.

### Western blotting analysis

Western blotting analysis was performed as previously described [21]. Embryos or oocytes were collected and washed 3 times in cold PBS and transferred in a maximum volume of 1.5 μL PBS into 1.5 μL of 2X extraction buffer supplemented with protease and phosphatase inhibitors (2X PBS, 2% (v/v) Triton X-100, 24 mM deoxycholic acid, 0.2% (w/v) sodium dodecyl sulfate, 20 mM NaF, 20 mM Na_4_P_2_O_7_, 2 mM PMSF, 3.08 μM aprotinin, 42 μM leupeptin and 2.91 μM pepstatin A – all from Sigma). The embryos were lyzed by three cycles of freezing in liquid nitrogen and thawing (with vortexing). Protein samples were diluted with 1 μL of 5X Laemmli buffer (50 mM Tris-HCl, 5 mM EDTA pH 8.0, 12.5% (w/v) sodium dodecyl sulfate, 0.05% (w/v) bromophenol blue and 25% beta-mercaptoethanol), incubated 10 min at 60°C and size separated using 20% homogenous SDS polyacrylamide gels (Pharmacia Sweden) on a PhastSystem apparatus (GE Healthcare, Castle Hill, NSW, Australia). Proteins were blotted into PVDF membranes (Hybond-P, GE Healthcare) in a semi-dry blotting apparatus overnight using transfer buffer (12 mM Tris PH 7.0, 96 mM Glycine and 20% (v/v) methanol). Nonspecific binding was blocked by 5% (w/v) skim milk in PBS supplemented with 0.05% (v/v) tween-20 (PBST) at room temperature for 1 h. Membranes were probed with primary antibody overnight at 4°C in 5% skim milk in PBST. A horseradish peroxidase conjugated second antibody was applied for 1 h at room temperature. Membranes were developed with either Pico SuperSignal Chemiluminescent Substrates (Pierce, Rockford, IL, USA) for 5 min at room temperature and exposed to X Ray film (CL-XPosure Film – Pierce).

Reprobing membranes with Lis-1 antibody allowed assessment of changes in levels of a constitutively expressed protein. Membranes were incubated in freshly made denaturing solution [7 M Guanidine Hydrochloride (Sigma), 50 mM Tris pH 8.0 (BDH), 2 mM EDTA (Sigma), 0.25% skim milk (Bonlac Foods Limited, Melbourne, Vic. Australia), 2 mM dithiothreitol (Promega Corporation, Madison, Wis., USA), 0.3% (w/v) BSA (CSL)] for 1 min at room temperature with mixing. Membranes were than rinsed twice with renaturation buffer [50 mM Tris pH 7.5 (BDH Laboratory Supplies, Poole, Dorset, England), 100 mM NaCl (Sigma), 2 mM EDTA (Sigma), 0.1% (v/v) tween-20 (Cat #P-7949 Sigma), 0.3% BSA (CSL)] and incubated with mixing in renaturation buffer for 30 min at room temperature. The membranes were than washed in PBST for 2 min to remove any remaining renaturation buffer, blocked and than re-probed with Lis-1 antibody. Primary antibodies were 1:200 anti-TRP53 (Ab-7) polyclonal antibody (Cat No: PC35, Oncogene Research Products, Merck, Kilsyth, Victoria, Australia) or 1:200 anti-Bax (P-19) polyclonal (Cat No: sc-526, Santa Cruz Biotechnology, Santa Cruz, CA.). TRP53 analysis was performed on groups of 30 embryos, for Bax the analysis was performed on the numbers shown in results.

Gels were digitally photographed and analyzed using LabWorks Image Acquistion and Analysis Software (Version 4.5.00.0; Ultra-Violet Products Ltd, Camdride, UK), using the area density tool. Bands were marked with the area of interest tool and optical density measured. Normalised optical density (OD, arbitrary units) of the p53 band, relative to its corresponding Lis-1 band was defined as the OD p53/OD Lis-1*100.

### Immunofluorescence

Embryos were washed 3 times in PBS with 0.1% BSA, 0.1% tween-20 and 0.2% (w/v) sodium azide (washing buffer), fixed with 2% paraformaldehyde (w/v) (Sigma) for 30 min and then permeabilized with 2% paraformaldehyde with 0.3% Tween-20 (Sigma) at room temperature for 30 min. Embryos were washed 3 times in washing buffer, blocked in 2% BSA and 30% serum for 3 h, and stained overnight at 4°C with primary antibodies: 1:300 anti-TRP53 (Ab-7) polyclonal antibody; or 1:100 anti-Bax (P-19) polyclonal (Cat No: sc-526, Santa Cruz Biotechnology). For each primary antibody an equivalent concentration of isotype control immunoglobulin was used as a negative control. Primary antibodies were detected by secondary antibodies coupled to FITC for 1 h at room temperature. Optical sectioning was performed with a Bio-Rad Radiance Confocal microscope, using a Nikon Plan Apo 60 X/1.4 oil emersion objective. Images were captured using Lasersharp 2000, Version 4.0 (BioRad). Microscope and laser settings were adjusted such that no fluorescence was observed with non-immune controls. A given antibody were observed with these same settings. All images were equatorial 1.5 μm optical sections generated by confocal microscopy. Greyscale images were converted to pseudocolor representation of staining intensity. Whole section imaging was performed with mercury lamp UV illumination and epifluorescence on a Nikon Optiphot microscope with an Olympus DPlan Apo 40 UV objective. Images were subjected to deconvolution using Image-Pro plus (Sharpstack, Media Cybernetics, Inc. Silver Spring, MD) and staining intensity represented by pseudocolor.

### Statistical analysis

Statistical analyses were performed with SPSS statistical package (Version 11.5, SPSS, Chicago, IL). The proportion of embryos developing to a given developmental landmark following culture was assessed by binary logistic regression analysis, treating the proportion developing to a given landmark as the dichotomous dependent variable and the treatments and experimental replicates as covariates in the model. The effects of treatments on the number of cells were assessed using univariate regression analysis within the General Linear Model.

## Results

**Figure 1:**
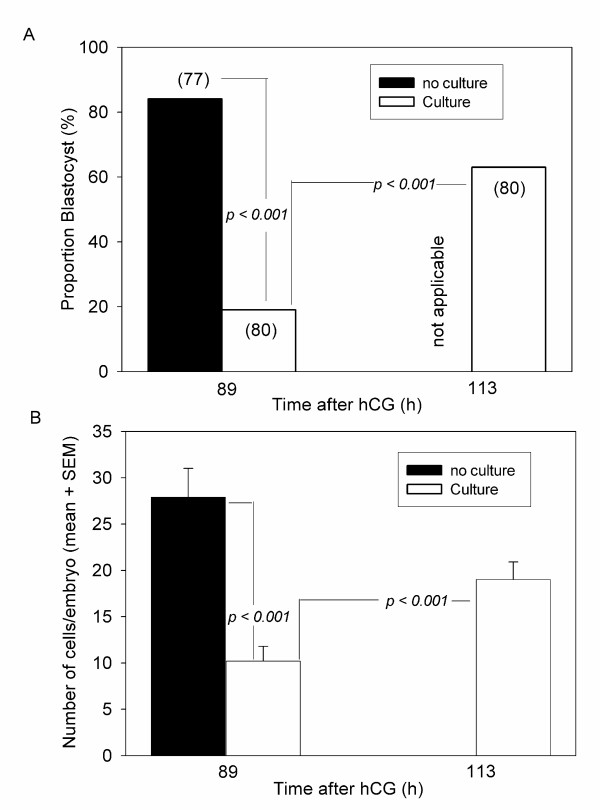
**The rate of development of cultured zygotes compared with embryos collected from the reproductive tract at the same time after the administration of ovulatory hCG (h)**. No embryos could be flushed from the uterus at 113 h since implantation had commenced (not applicable). Numbers shown in brackets are the number of embryos examined. (**A) **The proportion of embryos that were morphological blastocysts. (**B) **The total number of cells present in the blastocysts shown in Fig.1A (mean + SEM).

Cultured B6 zygotes developed at a significantly slower rate to the blastocyst stage than did equivalent embryos that developed in the reproductive tract (*p < 0.001*) (Fig. 1A). Most embryos were morphological blastocysts 89 h after hCG when development occurred in the reproductive tract but less then 20% when development was *in vitro*. This slower rate of morphological development was accompanied by a slower rate of cell proliferation. Embryos developing in the reproductive tract up to 89 h after hCG administration possessed around twice as many cells as those cultured (*p < 0.001*) (Fig. 1B). By 113 h after hCG those embryos developing in the reproductive tract had commenced implantation (and were not capable of being retrieved from the uterus by flushing), while those cultured *in vitro *were still retarded compared with the fresh 89 h blastocysts (*p < 0.01*) and still had fewer cells (*p < 0.01*) than 89 h blastocyst from the uterus (Fig. 1A).

**Figure 2:**
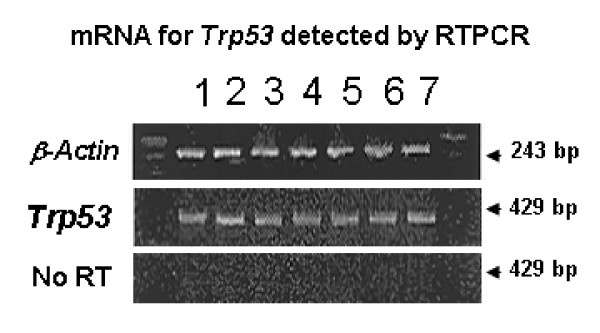
**Expression of *Trp53 *mRNA within preimplantation stage embryos**. RTPCR was performed with specific primers to detect β*-Actin *(positive control) or *Trp53*. The negative control (No RT) – *Trp53 *primers but no reverse transcriptase. 1. liver mRNA positive control, 2. oocytes. 3. zygotes. 4. early 2-cell stage. 5. late 2-cell stage, 6. morulae, and 7. blastocyst. Each analysis was performed on groups of 20 embryos and results are representative of 4 replicates.

**Figure 3:**
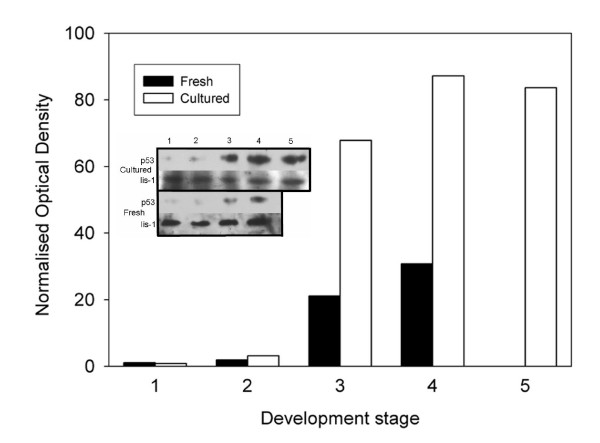
**Expression of TRP53 in embryos at various developmental stages**. Densitometric analysis of TRP53 expression as shown in inset. Optical density of p53 band/Lis-1 band *100. The numbers on X-axis represent – Cultured: embryos were collected at the zygote stage and cultured for: (1) 24 h, (2) 48 h, (3) 72 h, (4) 90 h. Fresh: were collected directly from the reproductive tract at the (1) zygote, (2) 2-cell, (3) morula, and (4) blastocyst stages. (5) A positive control for the expression of TRP53 was the analysis of ~1000 T47D breast cancer cells. **Inset **– Western blotting analysis of 30 embryos at various development stages. After analysis of TRP53 expression membranes were stripped and re-probed for expression of the constitutively expressed Lis-1 protein. The results are representative of 3 replicates. The numbers correspond to those on the graph.

Embryos expressed *Trp53 *mRNA at all preimplantation developmental stages examined (Fig. 2). TRP53 is generally considered to be constitutively expressed in most cell types, and its cellular concentration is largely regulated by its rate of degradation. Thus, an understanding of any potential role for TRP53 in embryo development is best analyzed at the level of protein expression. TRP53 expression was detected by Western blotting analysis in all preimplantation stages (Fig. 3). Densitometric analysis (Fig. 3) showed that for embryos collected directly from the reproductive tract, expression was at low levels at the pre-compaction stages. In morula stage embryos there was evidence of an increase in expression and expression was higher again in blastocyst stage embryos. For embryos cultured from the zygote stage there were similar levels of TRP53 expression during the pre-compaction stages as observed in embryos collected direct from the reproductive tract. At the morula stage expression was greater and in the blastocyst there was a marked increase in expression in cultured embryos compared with blastocysts collected fresh from the reproductive tract (Fig. 3).

**Figure 4:**
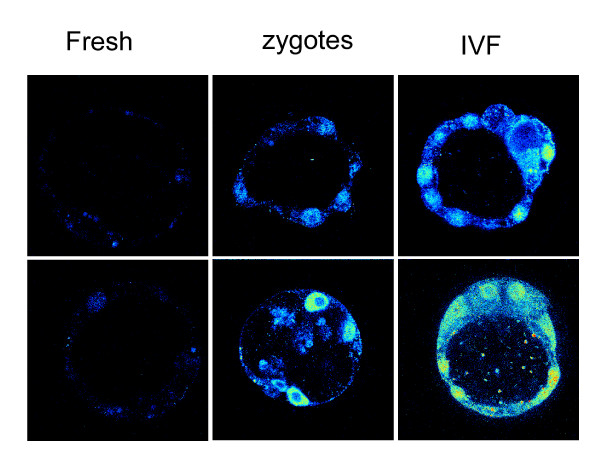
**Effect of IVF and culture on TRP53 localization in blastocysts**. Localization of TRP53 by indirect immunofluorescence in blastocysts collected from the uterus (Fresh); cultured from the zygote stage (zygotes); or cultured from the zygote stage after production by IVF (IVF). The plate shows images of 2 different representative embryos for each treatment. The experiment was repeated 3 times with a minimum of 8 embryos for each treatment in each replicate.

Immunolocalization showed that the increased TRP53 expression in cultured embryos was accompanied by a marked accumulation of TRP53 within the nuclei of some embryonic cells (Fig. 4). It was also shown that production of embryos by IVF followed by culture caused higher levels of TRP53 expression compared with culture of zygotes fertilized in the reproductive tract.

**Figure 5:**
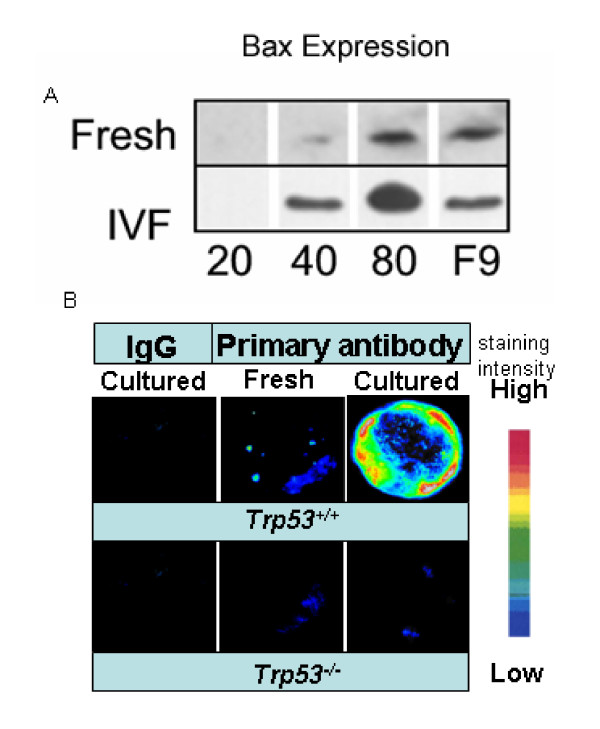
**TRP53 dependent expression of Bax in cultured blastocysts**.(**A**) Western blotting analysis using anti-Bax antibody of blastocysts collected from the uterus on day 4 (Fresh) or of embryos produced by IVF and cultured for 96 h (113 h post hCG). The embryos were extracted as groups of 20, 40 or 80 embryos and then subjected to Western blotting analysis. Approximately 500 F9 cells were also analyzed as positive controls. The blots shown are representative of 3 experiments. (**B**) The localization of Bax by indirect immunofluorescence (using same antibody as in Western blot analysis) with blastocysts collected from the uterus (Fresh) or those fertilized within the reproductive tract and the cultured for 96 h (Cultured) (113 h post hCG). Controls were subjected to IgG instead of primary antibody (IgG). The cultures were performed with *Trp53*^+/+ ^and *Trp53*^-/- ^embryos. Whole section images were deconvoluted and converted to pseudocolor representation of staining intensity. The sections shown are representative of 3 replicate experiments with a minimum of 8 embryos per treatment per replicate.

TRP53 is a transcription factor. It acts to promote transcription of many important proteins, including Bax. The increased TRP53 expression in cultured embryos was apparently transcriptionally active since Western blotting analysis showed that Bax was expressed at high levels in IVF and cultured embryos compared with blastocysts collected direct from the uterus (Fig. 5A). Whole embryo immunolocalization shows that the elevated expression of Bax occurred throughout the cultured blastocysts (Fig. 5B). Bax expression was not observed in cultured *Trp53*^-/-^*embryos *(Fig. 5B). This result demonstrates that the enhanced expression of TRP53 in cultured embryos was transcriptionally active.

**Figure 6:**
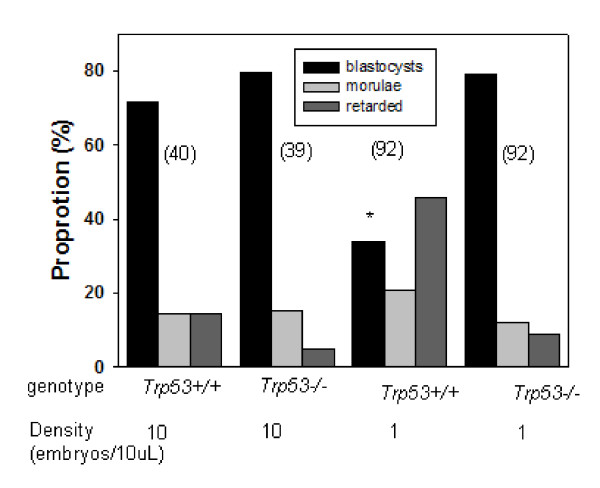
**The effect of *Trp53*^-/- ^*genotype *and the density of zygote culture on the development of zygotes *in vitro***. The results represent the proportion of zygotes that developed to morphologically normal blastocysts 113 h post hCG. The number of embryos in each treatment is shown in brackets. * shows a significant difference *p < 0.01 *compared with *Trp53*^-/- ^embryos.

Embryos are more susceptible to loss of viability in culture if they are cultured individually, compared with those cultured in groups. Figure 6 shows that *Trp53*^+/+ ^zygotes cultured individually had significantly poorer development to the blastocyst stage than did embryos cultured in groups of ten (*p < 0.001*). This adverse effect of individual culture was significantly (*p < 0.01*) alleviated in *Trp53*^-/- ^embryos. The results show that absence of *Trp53 *did not prevent the retarded development of embryos *in vitro *in groups, but did prevent the further retardation induced by culture at low embryo densities.

## Discussion

This study shows that culture of B6 zygotes resulted in their retarded development compared with equivalent age embryos developing in the reproductive tract. Embryos produced *Trp53 *mRNA throughout the preimplantation stage, confirming findings in other strains [12]. TRP53 protein was also expressed at relatively low levels during the normal preimplantation phase development. This low level of expression is consistent with the finding that TRP53 is not required for normal development of the embryo *in vivo*, since *Trp53*^-/- ^embryos are viable [22]. In blastocysts collected directly from the reproductive tract there is little obvious accumulation of TRP53 within nuclei and little expression of Bax, a TRP53 transcription product. These observations suggest a latency of expression and action of TRP53 in the embryo within the reproductive tract. Such latency is to be expected within normal cells [23]. By contrast, the culture of embryos from the zygote stage or their production by IVF (and subsequent culture) resulted in a marked increase in the expression of TRP53 in the post compaction embryos. This increased expression was accompanied by pronounced nuclear accumulation of TRP53. Culture also resulted in increased expression of Bax, and this was *TRP53*-dependent. Thus, TRP53 expression and nuclear localization in culture embryos was transcriptionally active. This result does not of itself show that Bax is a major effector of TRP53 expression in the cultured early embryo. However, the resistance of *Bax*^-/- ^mouse blastocysts (and partial resistance of *Bax*^+/- ^blastocysts) to apoptosis induced by the presence of high glucose concentrations [24], may suggest that it plays some role. TRP53 induces the expression of many genes and an important research question for the future will be to characterise the TRP53-induced transcriptome in the cultured early embryo in preimplantation stage viability.

Embryos that have *Mdm2 *activity deleted (*Mdm2*^-/-^) [15-17] show early lethality, and this was abrogated in the *Mdm2*^-/-^*Trp53*^-/- ^compound mutant. MDM2 acts as an E3 ubiquitin ligase that leads to ubiquitination and rapid degradation by the 26S proteasome of its targets, the most important of which is TRP53 [25,26]. The action of MDM2 is largely responsible for the short half-life of TRP53 in cells not subjected to stresses [23]. It is this degradation that accounts for the latency of TRP53 expression in most unstressed cells. MDM2 expression is in turn regulated by TRP53; the MDM2 promoter contains TRP53 consensus binding sites. Thus, MDM2 and TRP53 are recognised to form an autoregulatory feedback loop [27].

This study showed that the use of embryo's from mouse strains (such as the B6) that are susceptible to the various stresses of culture provide a useful model for the investigation of the cellular mechanisms of embryos response to culture. The use of this strain has the additional advantage that many gene knockout models are produced in the B6 background. The combined use of genetically modified animal models, experimental ART and modern cell biology provide the opportunity to ask fundamental questions about the embryos response to its culture environment.
